# Wearable EEG-Based Brain–Computer Interface for Stress Monitoring

**DOI:** 10.3390/neurosci5040031

**Published:** 2024-10-08

**Authors:** Brian Premchand, Liyuan Liang, Kok Soon Phua, Zhuo Zhang, Chuanchu Wang, Ling Guo, Jennifer Ang, Juliana Koh, Xueyi Yong, Kai Keng Ang

**Affiliations:** 1 Institute for Infocomm Research, Agency for Science, Technology and Research (A*STAR), 1 Fusionopolis Way, #21-01 Connexis (South Tower), Singapore 138632, Singapore; 2 Home Team Science and Technology Agency (HTX), 1 Stars Avenue, #12-01, Singapore 138507, Singapore; 3 College of Computing and Data Science, Nanyang Technological University, 50 Nanyang Avenue, Singapore 639798, Singapore

**Keywords:** Brain–Computer Interface (BCI), Cognitive Vigilance Task (CVT), Multi-Modal Integrated Task (MMIT), stress, distress

## Abstract

Detecting stress is important for improving human health and potential, because moderate levels of stress may motivate people towards better performance at cognitive tasks, while chronic stress exposure causes impaired performance and health risks. We propose a Brain–Computer Interface (BCI) system to detect stress in the context of high-pressure work environments. The BCI system includes an electroencephalogram (EEG) headband with dry electrodes and an electrocardiogram (ECG) chest belt. We collected EEG and ECG data from 40 participants during two stressful cognitive tasks: the Cognitive Vigilance Task (CVT), and the Multi-Modal Integration Task (MMIT) we designed. We also recorded self-reported stress levels using the Dundee Stress State Questionnaire (DSSQ). The DSSQ results indicated that performing the MMIT led to significant increases in stress, while performing the CVT did not. Subsequently, we trained two different models to classify stress from non-stress states, one using EEG features, and the other using heart rate variability (HRV) features extracted from the ECG. Our EEG-based model achieved an overall accuracy of 81.0% for MMIT and 77.2% for CVT. However, our HRV-based model only achieved 62.1% accuracy for CVT and 56.0% for MMIT. We conclude that EEG is an effective predictor of stress in the context of stressful cognitive tasks. Our proposed BCI system shows promise in evaluating mental stress in high-pressure work environments, particularly when utilizing an EEG-based BCI.

## 1. Introduction

### 1.1. Quantifying Stress with a BCI System

Stress is a common feature in high-pressure environments with high cognitive loading, such as air traffic control centers, emergency departments, and call centers [1,2,3]. In the short term, stress can result in positive, negative, or complex effects on task performance, depending on the specific tasks and individuals in question [4,5,6]. In the long term, however, stress is known to adversely affect physical and mental health, and chronic stress is causally associated with a variety of disease states [7,8]. As people are over-exposed to cognitively demanding tasks, they can suffer from stress, as well as other factors that impair performance [9,10,11].

Mental stress is traditionally measured by subjective self-reports, or through objective biomarkers such as heart rate variability (HRV), skin conductance, blood pressure, pupillary response, and cortisol levels [12,13]. For a given individual, subjective stress and objective stress are highly correlated with each other, and subjective stress may be a better predictor of stress-related health issues [14,15,16]. Nonetheless, subjective stress must be measured by surveys or questionnaires, and it is impractical for people working in a high-pressure job to regularly fill in a form to measure their subjective stress. In contrast, objective stress can be automatically recorded by wearable devices while not interrupting peoples’ work.

For this project, we aim to improve mental stress quantification by developing a BCI system that can automatically and objectively measure mental stress using wearable devices. We chose to use EEG as our primary recording method, as EEG is well-established as a non-invasive BCI medium, being able to robustly and accurately record neural signals that correlate with motor imagery, affective states, epileptic seizures, and other factors [17,18,19]. Therefore, we conducted a lab trial using EEG recordings while participants performed stressful cognitive tasks to collect data for developing models of mental stress. Another important consideration of the requirements of this BCI system is the ease of use and portability. As such, wet electrode-based electroencephalogram (EEG) systems (involving the use of a conductive gel between the electrode and the scalp to record brain signals) are not suitable for this use case. Instead, a commercially available dry electrode EEG headband, the Neeuro Senze band, was evaluated, and was found to be more appropriate. This device is non-invasive, does not require the use of wet gel for operation, is easy to wear and remove, and is readily wearable by participants for the detection of stress. (See [20] for a review on dry EEG electrodes). To complement the EEG recordings, we also used an electrocardiogram (ECG) monitor, which recorded heart rate and heart rate variability, both well-established physiological markers that can indirectly quantify stress.

To build the BCI computational model, we developed the Multi-Modal Integration Task (MMIT) to better reflect the working environment and stressors that an officer in a command-and-control center may face. The MMIT was based on the video game “Papers, Please”, in which the player takes the role of an immigration officer who is required to rapidly and accurately accept or reject people at a checkpoint [21]. This game is noted for its stressful gameplay, and has been discussed in publications involving philosophy and politics [22,23]. During the MMIT, participants are presented with procedurally generated suspects with several properties and are tasked to match them against a set of rules. The MMIT requires participants to sort through different categories of information to make a decision while under limited time, and time pressure is known to be a stressor [24].

In addition, we challenged our participants with the Cognitive Vigilance Task (CVT), an extant psychological task that involves reading sets of digit pairs and pressing a button if one pair meets a certain condition. The CVT has been validated by many studies as a task that demands cognitive engagement [25,26]. By challenging our participants with both a conventional CVT approach as well as a novel MMIT approach that is tailored to simulate a more realistic stressful working environment, we were able to obtain the advantages of both.

To quantify the psychological and physiological correlates of stress during the MMIT and CVT, we recorded EEG signals, ECG signals, written survey results, and computerized test results from participants, with the goal of developing a BCI model for detecting stress. The lab trials were conducted from 1 August 2023 and concluded on 6 October 2023, recruiting a total of 40 participants. All experimental procedures were approved by the A*STAR Institutional Review Board (IRB) prior to recruitment (A*STAR IRB reference: 2023-055).

### 1.2. Novelty and Contributions

There are many extant studies on quantifying stress by EEG. Nonetheless, from the outset, we aimed to design and complete an investigation with specific goals and methods that distinguish it from the others.

Firstly, the vast majority of contemporary EEG-based stress studies induce stress via the Stroop Color and Word Test (SCWT), Montreal Imaging Stress Task (MIST), or Trier Social Stress Test (TSST) [12,13,27,28]. The SCWT and MIST are similar to the CVT we implemented in that they are several-second-long cognitive tasks. In contrast, the TSST involves public speaking, and is designed to induce psychosocial stress rather than cognitive stress. For this study, we aimed to create a stress task that simulated the heavy cognitive burdens of people performing high-pressure jobs. This is why we created the MMIT, which can be seen as an extension of the SCWT, but instead of requiring participants to memorize and apply only one condition (name the color of the stimulus) to the stimuli appearing during each trial, the MMIT includes multiple conditions which must be evaluated simultaneously.

Secondly, we designed an experimental protocol that simulates the burdens of working in a stressful job for prolonged periods of time. Regular day-jobs often require people to carry out their work for several hours without breaks, and people who work shifts, such as paramedics or police officers on patrol, may even need to maintain their vigilance for even longer during busy periods. In contrast, most EEG-based stress studies we found tend to have far shorter task durations, ranging from 15 to 19 min in total per participant [27,28,29]. While such short-duration tasks are easier to organize for both experimenters and subjects, we believe that they may be less suited for simulating stressful jobs. Hence, we designed a protocol that was 140 min long, and required participants to perform each of the two tasks for 40 min without a break.

Thirdly, our ultimate goal is to create a portable system that can detect stress in working people as they are doing their jobs. Unfortunately, this requirement excludes the use of full-head-cap wet EEG electrodes, which are the gold standard for EEG recordings and ubiquitous in scientific studies [12,13], but are bulky, difficult to put on, and are generally tethered by cables to bulky recording equipment. For similar reasons, we decided not to record other common physiological correlates of stress, such as electrooculography, cortisol levels, electrodermal activity (EDA), or blood pressure [12,13], as they would increase the scientific value of the study, but decrease the odds of end-user adoption and compliance with the stress-detection system.

There was also one study we found that used dry EEG recordings to detect stress states to a very high level of accuracy (99.98%) [29], however, it used subject-specific models, and is likely to be a case of overfitting. Instead, we used a dry four-electrode headband, which working adults can feasibly wear by themselves to monitor their stress levels. Also, we evaluated our models in a leave-one-subject-out manner, thus generating stress classification models that are more applicable to the general population rather than just a single participant.

## 2. Materials and Methods

### 2.1. Inducing Stress

While there are several ways that researchers can impose stress on human participants, the threat of social evaluation has been most established in lab studies to elicit stress [30]. Some common stress induction methods include the Trier Social Stress Test (TSST) [31,32], where participants are instructed to deliver a speech, and the Sing-a-Song Stress Test (SSST), where participants are made to sing a song in the presence of another person [33,34]. Social evaluation tasks induce stress due to the risk of embarrassing oneself in front of another person.

Other stress induction protocols impose stress by physical means, such as the cold pressor test, where participants have a hand immersed in icy water [35,36], and the carbon dioxide test, where participants breathe air containing 35% CO_2_ [37,38]. Some stress induction paradigms even combine physical and psychological stress, such as the Socially Evaluated Cold-Pressor Test (SECPT) [39] and the Mannheim Multicomponent Stress Test (MMST) [40].

A third broad category of stress tests encompasses cognitive tasks, like the Stroop Color and Word Test (SCWT) where participants are required to exercise inhibitory control [41], and the Cognitive Vigilance Task (described further in Section 2.2.1) [25,42]. Cognitive tasks impose a heavy mental load on participants and require them to deeply engage with the task to successfully complete trials.

Ultimately, we decided not to use a task that imposes the threat of social evaluation alone. Any psychological task in a laboratory setting inherently imposes some degree of social threat due to the presence of the experimenter performing the evaluation, and for a stress task to be ecologically valid (i.e., relevant to the target population), it should expose participants to multiple sources of stress [43]. On the other hand, physical stress induction protocols like the cold pressor test and CO_2_ challenge test offer their own challenges: bringing buckets of icy water into a lab full of electrical equipment is a safety hazard without special measures to prevent water spillage [44], and CO_2_ inhalation requires specific equipment.

Thus, we adapted a cognitive stress induction task from vigilance research, the CVT. Additionally, we designed a second task that is challenging for participants and includes adaptive difficulty levels, the MMIT (described in Section 2.2.2). These do not require special equipment beyond a regular desktop computer, and the cognitive stresses they generate are similar to the kinds of stress that people face in high-pressure work environments, enhancing ecological validity [43].

### 2.2. Cognitive Tasks

Participants were instructed to perform two computer-based tasks, the Cognitive Vigilance Task (CVT) and the Multimodal Integration Task (MMIT). Both tasks (lasting 40 min each) are cognitively demanding and are designed to induce stress in the participants. During both tasks, participants wore a non-invasive electroencephalogram (EEG) headband to record neural signals, as well as an electrocardiogram (ECG) monitor to record their heart rate (HR) and heart rate variability (HRV).

#### 2.2.1. Cognitive Vigilance Task (CVT)

Maintaining vigilance over sustained periods induces stress as vigilance is both unstimulating yet mentally demanding [45,46]. During vigilance tasks, participants often report feeling stressed, as characterized by physiological measurements and self-reports [45]. Thus, we adopted the Cognitive Vigilance Task (CVT), an established paradigm in vigilance research where participants monitor the occurrence of critical signals on a screen, and employed a modified version [25,42]. In the CVT, participants were required to detect critical signals: two-digit numbers in which the first and second digits differ by either 0 or 1.

**Figure 1:**
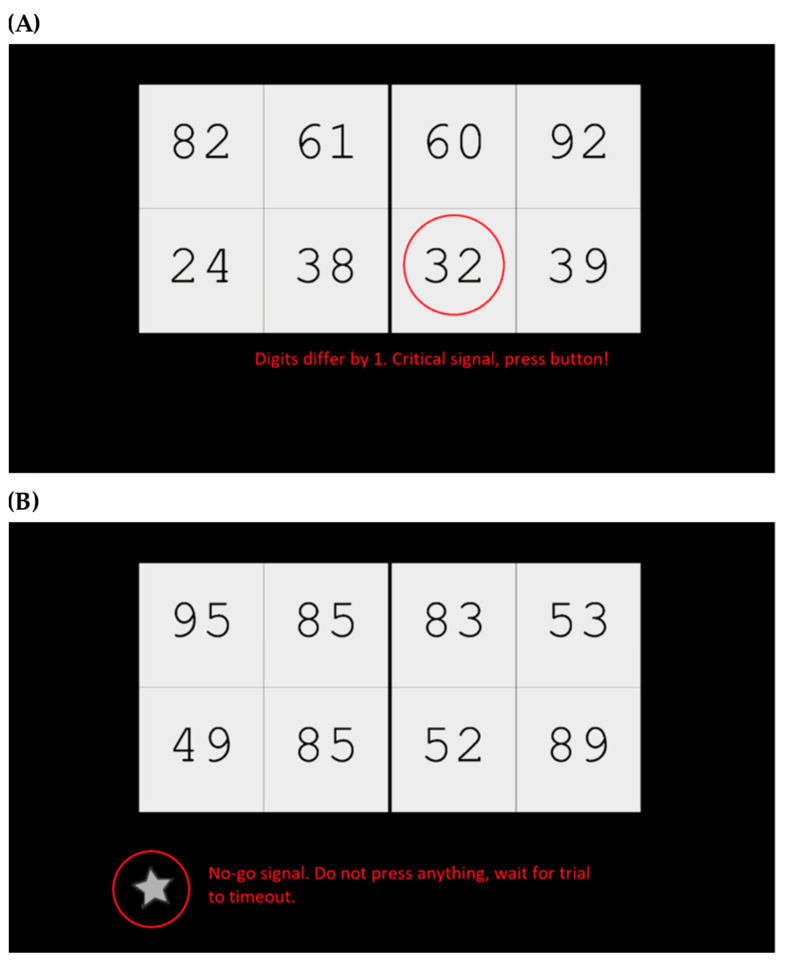
Cognitive Vigilance Task. (**A**) A critical number, 32, is shown in this trial. The number 32 is critical, as the digits differ by 1. The other numbers are not critical, as their digits do not differ by 0 or 1. Comparisons between numbers in different squares (e.g., 61 and 60) are not relevant in this task, and do not constitute a critical number. (**B**) A no-go trial. In no-go trials (which all included a critical number, in this case 89), a white star appeared at the bottom left of the screen. Participants were required to wait for trial to time-out. For both screenshots, the red annotations did not appear to the participants during the actual task.

The CVT we used can be seen in Figure 1. For each trial, a panel of 8 two-digit numbers was shown, and participants were required to detect critical numbers which occurred infrequently, either in only 1 of the 8 numbers, or not at all. Participants were instructed to press the spacebar if a single critical number was present (positive trial), and to press nothing and let the trial time-out if there were no critical numbers (negative trial).

Additionally, we integrated a go/no-go task into the CVT to elevate its complexity and cognitive load, necessitating participants to exercise inhibitory control [47]. Throughout certain trials, participants encountered a visual no-go cue represented by a gray star (refer to Figure 1). These cues were presented selectively alongside a critical number. Participants were instructed to refrain from pressing any button and allow the trial to time-out when presented with the no-go cue.

Participants were required to perform the CVT continuously for 40 min, during which we presented blocks of trials with unvarying difficulty, and a constant-length time window (2.5 s) for responding. Thus, the CVT was designed to be repetitive yet cognitively demanding; in other words, stressful [9].

#### 2.2.2. Multi-Modal Integration Task (MMIT)

The Multi-Modal Integration Task was inspired by the video game “Papers Please” [21], which puts the player in the role of an immigration officer who must decide whether to admit or deny the entry of procedurally generated characters into a fictional country, based on whether their documents complied with a set of immigration rules. The game has been noted for its ability to impose complicated cognitive burdens and time pressure onto players, and time pressure is a known stressor [24]. Nonetheless, existing publications on the game have focused on the ethical and sociopolitical implications of the game narrative, rather than how the gameplay can induce stress in players [22,23]. Our experiment sought to explore the neuropsychological effects of decision-making under pressure, creating an engaging and immersive task for participants.

In the MMIT, participants were required to match properties of a procedurally generated suspect (displayed in the box on the right) against a list of given rules (displayed on the left of the screen). Participants were instructed to press the spacebar if all rules matched (positive trial), and if not, to wait for the trial to time-out (negative trial). A no-go sub-task was also present: if a gray star appeared on the screen when all rules matched, participants were instructed to let the trial time-out (no-go trial). As with the CVT, the no-go task increased the complexity of the task by requiring the participants to practice inhibitory control [47].

**Figure 2:**
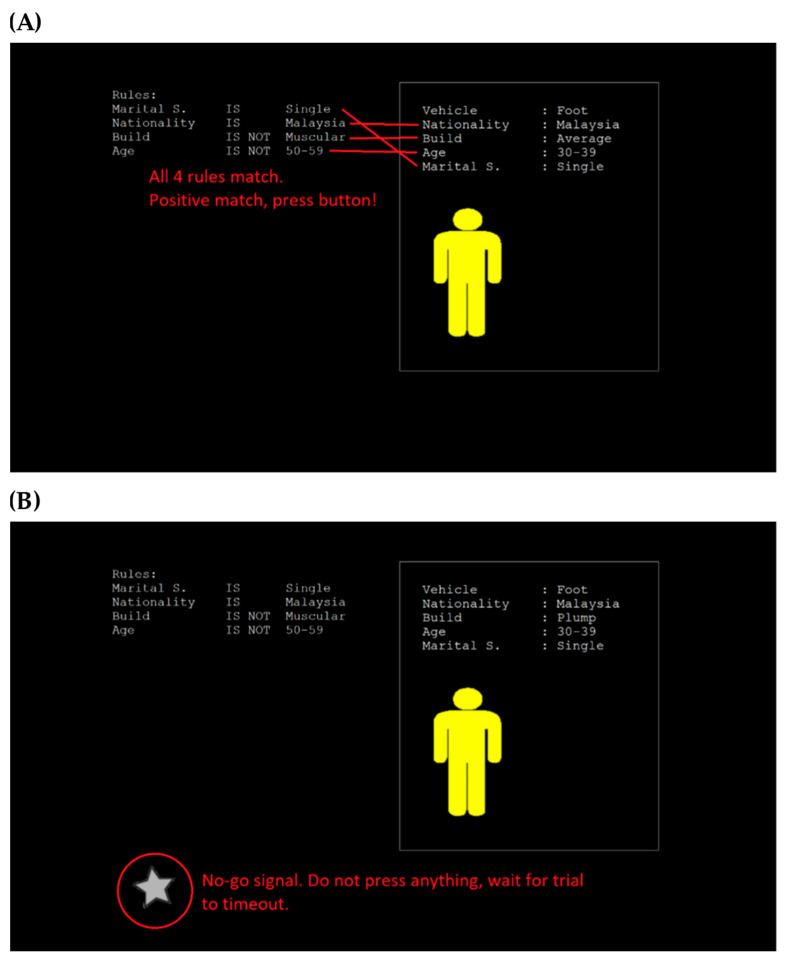
Multi-Modal Integration Task. (**A**) This screenshot shows a trial in which all properties of the suspect match the rules, meaning that the participant should respond by pressing the spacebar. (**B**) This screenshot shows a no-go trial, meaning that the participant should respond by waiting for the trial to timeout. For both screenshots, the red annotations did not appear to the participants during the actual task.

Trials were presented in blocks of 10, where each block contained 6 positive trials, 2 negative trials, and 2 no-go trials. Like the CVT, after each block of 10 trials, the results were briefly presented to participants, describing how many trials out of 10 they had responded correctly to (or correctly allowed to time-out for the negative and no-go trials). Participants were not given additional information on the breakdown of their score. Below, in Figure 2, are screenshots of a sample go-trial and a sample no-go trial, with annotations in red for instructional purposes.

Participants were required to do the MMIT continuously for 40 min. However, unlike the CVT, the MMIT trials were of variable length. Initially, the experiment started with a trial duration of 5 s. For each block of trials, when a participant performed “well”, that is, if they responded to 9–10 trials out of 10 correctly, the difficulty level was raised by decreasing the trial duration by 1 s (to a floor of 1 s), giving the participant less time to interpret each trial and respond appropriately. Conversely, for each block of trials where the participant performed “badly”, that is, responding to 7 or fewer trials out of 10 correctly, the difficulty was lowered by increasing the trial duration by 1 s, giving the participant more time per trial. If they scored exactly 8 out of 10, the trial duration remained unchanged.

By making trial length variable, we aimed to implement adaptive difficulty for this task, so participants would always be cognitively challenged. This procedure would also ensure that participants with different levels of cognitive ability and/or reaction times would all experience similar levels of stress.

The adaptive difficulty component also promoted the continued engagement of each participant. During our internal testing before the experiment proper (data not presented), we found that different individuals had a wide range of response times when performing the MMIT, so we implemented variable difficulty, allowing the task to adapt to individuals with different response times, instead of using a single trial length, which would have been too short for some people and too long for others.

### 2.3. Self-Report of Stress

To measure the self-reported stress levels experienced by the participants, we administered a modified Dundee Stress State Questionnaire, a questionnaire that is designed to measure 3 stress states: task engagement, distress, and worry [48,49]. Task engagement is related to focus/concentration/interest in the task, distress is a negative emotion related to the perception of not having control, and worry relates to experiencing distracting levels of metacognition [2]. We decided to measure both objective physiological correlates of stress, as well as subjective self-reported levels of stress, as these factors can interact in complicated ways and do not necessarily match each other [50]. We administered the modified DSSQ thrice to each participant, to record their reported stress levels before and after each task (more details are available in Section 2.5.1).

### 2.4. Task Performance Metrics

For both the CVT and MMIT, we recorded two scores related to task performance: the proportion of correct and wrong responses per block (accuracy), and the mean response time for positive trials (reaction time). Reaction time only makes sense if a trial is positive and the participant responded correctly; trials where the participant pressed the spacebar when it was a negative or no-go trial, or trials which were timed out for any reason, were excluded from reaction time calculations. For the MMIT, there was a third task score, trial length. As mentioned above, if a participant performed well, the trial length would adapt by decreasing, while if they performed poorly, the trial length would adapt by increasing.

### 2.5. Design of Experimental Sessions

In each experimental session, there were 5 sub-sessions: Resting (Baseline), CVT practice, MMIT practice, CVT and MMIT. A total of 40 participants were recruited in this study.

#### 2.5.1. Sequence of Events

Since each participant had to perform the CVT once and the MMIT once, our experimental results may have been confounded by order effects [51]. To mitigate these, we completely counterbalanced our participants. Half the participants were randomly assigned to do the CVT first followed by the MMIT, while the other half were assigned to do the MMIT first and then the CVT. Data from the two groups was then pooled together during analyses.

At three points (steps 2, 5, and 8 in Table 1), participants were instructed to fill in a short questionnaire, a modified Dundee Stress State Questionnaire [49], to quantify their subjective stress state. A flowchart of the experimental protocol is shown in Table 1:

#### 2.5.2. Experimental Timeline and Participants

Participants were recruited from the A*STAR, Neeuro, and HTX communities. In total, we recruited 40 participants. Participants were required to be healthy adults between the ages of 21 and 65. We recruited a balanced ratio of male and females: 17 were male and 23 were female. Recordings were performed over a 2-month period from 1 August 2023 to 6 October 2023.

**Figure 3:**
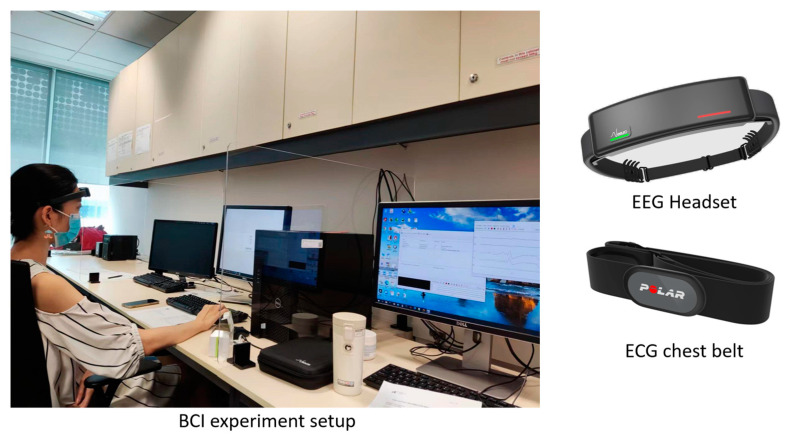
Example of the experimental setup. The right-side screen enabled the operator to monitor the EEG/ECG signals and data acquisition process. The left-side screen was for displaying visual cues to the participant for the cognitive tasks. Each participant wore an EEG headband on their forehead, and an ECG chest belt.

All experiments were conducted at the Institute for Infocomm Research, Singapore. The experimental setup was run on a desktop running Windows 10. There were separate monitors for participant and experimenter. Figure 3 shows the experimental setup.

### 2.6. Recording and Processing ECG Signals

**Figure 4:**
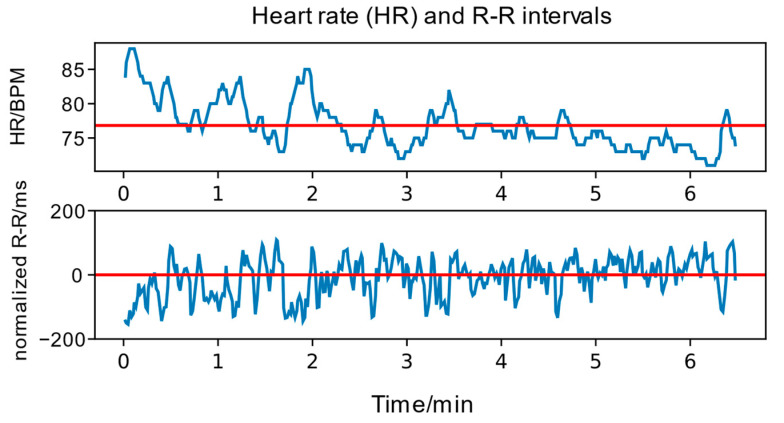
Heart rate (HR) and R-R interval plots for one participant for one short practice session (7 blocks). HR is measured in beats per minute (BPM), and the R-R interval is measured in milliseconds (ms). The R-R interval is normalized to zero mean. The red line is the mean HR and R-R interval, respectively.

We used ECG recordings to derive heart rate variability (HRV) measurements, which can be used as a measure of objective stress [12,52]. The Polar ECG device produced an HRV measurement in the form of R-R intervals. Below, Figure 4 shows an example of the HRV parameters extracted from ECG recordings.

The HRV measurements thus were recorded as a periodic list of R-R interval values. This was converted to a .txt file. Due to inevitable instances of signal loss and harmless ectopic heartbeats in the adult heart [53], the list of R-R values was dotted with Not a Number (NaN) values, indicating missing data points.

Thus, we applied the following preprocessing steps to extract HRV features:

1. Remove outliers in the list of R-R intervals (R-R intervals that are lower than 300 ms or greater than 2000 ms).
2. Replace NaN values via linear interpolation.
3. Remove ectopic beats using the Malik method [54]. This gives the N-N interval, which now contains NaNs for the removed ectopic beats.
4. Replace NaN values via linear interpolation.

This preprocessing produced an interpolated N-N interval with no NaNs. From these interpolated N-N intervals, we computed HRV features across each block of trials. Each block was typically around 1–1.5 min, and this duration was sufficient to obtain accurate and stable HRV features.

We calculated these HRV features using open-source libraries for HRV analysis [55]. The HRV features are listed in Table 2. These HRV features are standardized and established for HRV analysis (see [56]).

### 2.7. Recording and Processing EEG Signals

EEG signals were acquired from Senzeband 2 (Neeuro Pte Ltd., Singapore), an EEG headset equipped with 4 recording channels, located at positions Fp1, Fp2, T9, and T10. In addition, there is a reference electrode at FpZ. An elastic strap was adjusted to maintain a good fit over different head sizes. Before recordings started, the experimenters rearranged the strap and electrodes until impedance levels were between 10 kΩ and 100 kΩ.

The raw data were transmitted via Bluetooth BLE 5 to the server. Throughout the experiment, all data were consistently stored in binary format, facilitating subsequent offline analysis. To ensure data quality, EEG signals underwent bandpass filtering between 0.5 Hz and 40 Hz, effectively eliminating unwanted frequency components and noise. Subsequently, two bipolar signals, namely T9-Fp1 and T10-Fp2, were selected for further analysis and modeling. Bipolar derivations offer distinct advantages over unipolar setups, including enhanced spatial resolution and noise reduction. By measuring the voltage difference between two electrodes, bipolar configurations provide more localized and precise neural activity measurements, thus contributing to more accurate modeling outcomes.

Despite meticulous preparation, the nature of dry EEG sensors introduced challenges, leading to poor data quality for several participants. Post-experiment manual inspection revealed instances of flatline EEG signals, indicating that the signal was lost for that time. Consequently, data from 5 participants were excluded from EEG analysis due to the presence of such artifacts. The remaining dataset from 35 participants formed the foundation for subsequent modeling and analysis, enabling us to draw meaningful conclusions regarding the neural correlates under investigation.

### 2.8. EEG Feature Extraction

EEG recordings were divided into blocks of 10 trials, and features were extracted from each block. Each block typically lasted 1–2 min for MMIT (depending on the participant’s level), and approximately 1.5 min for CVT. Given that features were computed independently for each channel, there were n × m EEG features for every trial, where n represents the number of channels and m indicates the number of different EEG features. We used bipolar EEG signals for feature extraction, where ch1 = TP10-FP2, and ch2 = TP9-FP1. For each channel, the EEG signal is *X*(*i*), *i* = 1,2,…N, where N is the sample number of a moving window of 2 s.

Two groups of EEG features were extracted for modeling purposes. Firstly, power in specific frequency bands, known as power spectral density (PSD), was computed. These bands included delta (0.5–4 Hz), theta (4–8 Hz), alpha (8–13 Hz), beta (14–30 Hz), and gamma (>30 Hz). The absolute power of each band was determined by averaging the power within the frequency range. Furthermore, the relative power of each band was calculated as the ratio of its absolute power to the total power across all frequency bands.

Non-linear features were also extracted from the EEG data, including sample entropy [57], Fractal Dimension [58], and Hjorth parameters [59]. Electrophysiological dynamics are inherently non-linear, and non-linear features are thought to represent them better [60,61]. An entropy analysis on time-series data enables a statistical quantification of the level of uncertainty or randomness in the patterns, reflecting the amount of information contained in the signal. For instance, during anesthesia, a significant decrease in EEG entropy implies that the signal becomes more predictable or repetitive as anesthesia takes effect [62]. We also examined Fractal Dimension (FD), a measure of signal complexity and self-similarity in EEG signals that is related to emotional states and neural dynamics in the brain [63,64,65]. Furthermore, we extracted Hjorth parameters, including Hjorth Activity, Hjorth Complexity, and Hjorth Mobility. These features have been reported to be associated with emotion states [66,67]. Details on deriving these non-linear features are described below.

#### 2.8.1. Sample Entropy

Sample Entropy (SampEn) [62] is a measure of the complexity or irregularity of a time-series signal. Given an EEG signal *x*(*i*), 1 < *i* < *N* of length *N*, construct vectors of length *m*:

(1) $$\;\;\;X_{m}\left(i\right)=\left[x\left(i\right),\;x\left(i+1\right),\;\ldots ,\;x\left(i+m-1\right)\right]\;for\;i=1,\;2,\;\ldots ,\;N-m+1$$

where *m* is the embedding dimension. *N* is the length of *X*(*i*). Calculate the distance between two vectors *X*(*i*) and *X*(*j*) using the maximum norm:

(2) dXi, Xj=maxk=0 to m−1⁡ xi+k−xj+k

For each *i*, count the number of *j*, such that *d*[*X*(*i*), *X*(*j*)] ≤ *r*. Denote this count as $B^{m}(i).$ Then, compute the probability:

(3) $$\;\;\;C_{i}^{m\;}\left(r\right)=\left(\frac{1}{N-m+1}\right)\sum_{i=1}^{N-m+1}\frac{B^{m\;}(i)}{N-m}$$

Repeat the above steps for vectors of length *m* + 1 to compute $C^{\left\{m+1\right\}}\left(r\right).$ Finally, Sample Entropy is defined as:

(4) $$SampEn(m,\;r,\;N)=-ln(\frac{C^{\left\{m+1\right\}\;\;}\left(r\right)}{C^{m}\left(r\right)})$$

This value quantifies the predictability of the signal.

#### 2.8.2. Fractal Dimension

The Fractal Dimension (FD) is a measure that quantifies the complexity of an object or signal by examining how the details of the object change with scale. In the EEG analysis, FD provides a measure of the signal’s structural complexity over time.

The formula for calculating the Fractal Dimension using the Higuchi method [58] is given by first calculating the length of the curve *L*(*k*) for a segment size *k*, which is computed as:

(5) $$L(k)=\frac{1}{k}\sum_{i=1}^{k}\left(\frac{\left|X_{\left(i+k\right)}-X_{\left(i\right)}\right|}{k}\right)$$

The Fractal Dimension (FD) is estimated by plotting *log*(*L*(*k*)) versus *log*(*k*) and fitting the slope of the line that fits these points. The higher FD values indicate more complex, irregular brain activity, while lower FD values suggest more regular, predictable patterns.

#### 2.8.3. Hjorth Activity and Hjorth Complexity

The Hjorth parameters, Activity and Complexity, are time-domain features used to describe the characteristics of an EEG signal [59], which provide insight into the signal’s statistical properties. The formulas and descriptions for both features are detailed as follows:

Hjorth Activity is a measure of the signal’s variance or the power of the signal.

(6) $$\ddot{A}=Var\left(X_{i}\right)=\frac{1}{N}\sum_{i=1}^{N}{\left(X_{i}-\mu x\right)}^{2},\;0<i<N$$

where $X_{i}\;$represents the EEG signal, μx is the mean of $X_{i}$, and $Var(X_{i})$ is the variance of the signal. It provides a basic estimate of the signal’s amplitude variation over time. A higher Hjorth Activity value indicates more intense fluctuations in the EEG signal.

Hjorth Complexity is a measure of the signal’s change in frequency. It reflects how the shape of the EEG signal changes over time in relation to its baseline (Activity $\ddot{A}$). It is related to the frequency content of the signal and can be considered as a normalized measure of the curvature of the signal.

(7) $$\ddot{C}=\frac{\sqrt{Var(\Delta X_{i})}}{\sqrt{Var(X_{i})}}$$

A higher Complexity $\ddot{C}$ value suggests a more irregular or faster-changing signal.

### 2.9. Computational Models for Stress Classification

**Figure 5:**
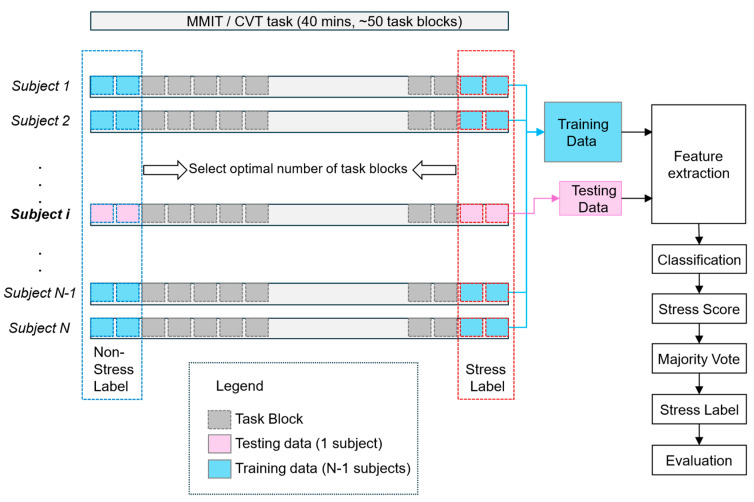
Diagram of our stress detection algorithm, indicating how it was trained and tested. The blue dashed box indicates the blocks that were labelled as “non-stressed”, and the orange dashed box indicates the blocks that were labelled as “stressed”.

**Figure 6:**
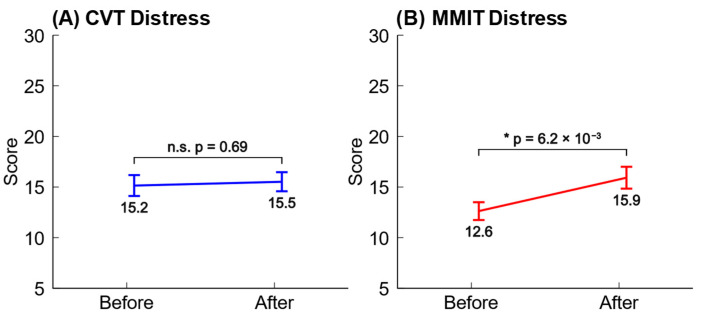
Changes in subjective stress levels before and after the CVTs and MMITs. Error bars represent the standard error of the mean, and the significance levels were calculated using paired two-tailed *t*-tests (α = 0.05). (**A**) Performing the CVT did not significantly increase the reported levels of distress. (**B**) Performing the MMIT significantly increased the reported levels of distress.

Both the CVT and MMIT are intentionally designed to provoke stress responses. We posit that the beginning of the task session can be characterized as a “non-stress” state, while its ending part represents a “stress” state. This hypothesis is substantiated by the DSSQ self-reported mental state assessment (See Figure 5). Consequently, each participant contributed two distinct sets of EEG data: one from the CVT and the other from the MMIT, both labeled for supervised stress classification. As depicted in Figure 6 below, this approach enabled us to identify the optimal number of task blocks for constructing the training dataset for optimal stress detection. To assess the effectiveness of our approach, we employed a leave-one-subject-out validation methodology. This involves partitioning our data into training and testing sets using an N−1 versus the remaining one participant split, ensuring a comprehensive evaluation and validation of our algorithms’ performance.

For each model, we used a Support Vector Regressor model to derive a stress score from the input features, subsequently employing a majority voting mechanism to ascertain the stress states of the task block. This voting system assesses the entirety of each block by examining its epochs. If more than half of the epochs within a block indicated stress, the overall state of the block was classified as “stressed”; otherwise, it was categorized as “not stressed”. We then trained an SVR classifier on all but one participant and tested it on the left-out participant. This process was repeated for every participant.

In order to reduce non-stress-related noise contained in the EEG data, we adapted another method that was previously used to decode fatigue using EEG [68]. This method entails identifying the task blocks most strongly associated with the feature being decoded (in this case stress), and instead of using the full dataset, only using those blocks for training and evaluating models.

## 3. Results

In this section, we report the results and trends from (1) self-reported stress levels, (2) task performance and (3) the grouped analysis of EEG and ECG measurements.

### 3.1. Analysis of Dundee Stress State Questionnaire (DSSQ) Responses

For the purposes of this study, we focused on the distress factor in the DSSQ, using it as a measure of stress. Higher scores of distress indicate higher stress levels, and it has been previously published that stressful tasks cause more significant changes in distress than engagement or worry [2]. We found that the CVT and MMIT had very different effects on the participants’ subjective stress levels. Performing the CVT caused participants to report no significant changes to their distress, while performing the MMIT caused participants to report significantly higher distress. The mean changes in distress across participants are plotted below in Figure 5.

Based on the empirical results from our survey data, we decided to label the data from the start of the MMIT and the end of the MMIT as “not stressed” and “stressed”, respectively.

### 3.2. Stress Level Predictions

**Figure 7:**
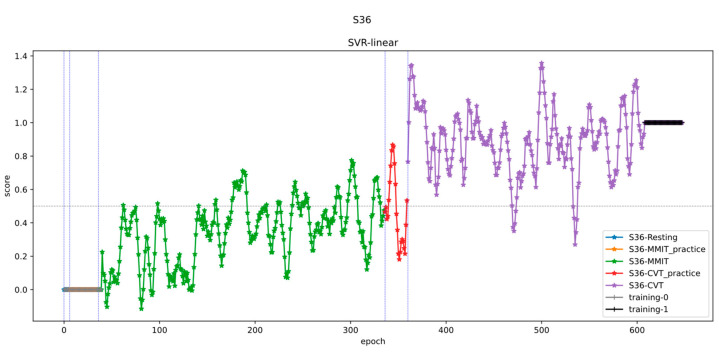
Predicted stress levels of a participant as they performed the CVT and MMIT. The green points represent task blocks recorded during the MMIT, and the purple points represent task blocks recorded during the CVT. There was an overall trend towards higher predicted stress levels; however, the block-to-block variability was considerable.

Here, we show the results of a model we trained as described previously to predict stress of a subject over the course of an experimental session, including both the CVT and MMIT sessions (Figure 7). The participant’s predicted stress levels increased over the course of the experiment. Their stress scores increased from start of first task to end of first task (MMIT) and remained high during second task (CVT). This is in line with our expectations, as the two tasks are designed to be stressful with tight time constraints and a high cognitive load.

Nonetheless, we noticed that there was a high degree of variability between the predicted levels of stress between blocks. We considered the possibility that this variability was caused that noise that could be smoothed out by increasing the number of blocks used to train each model; hence, we experimented with using different numbers of blocks for model training (see Section 3.3 below).

### 3.3. Performance of Stress Classification Models

**Figure 8:**
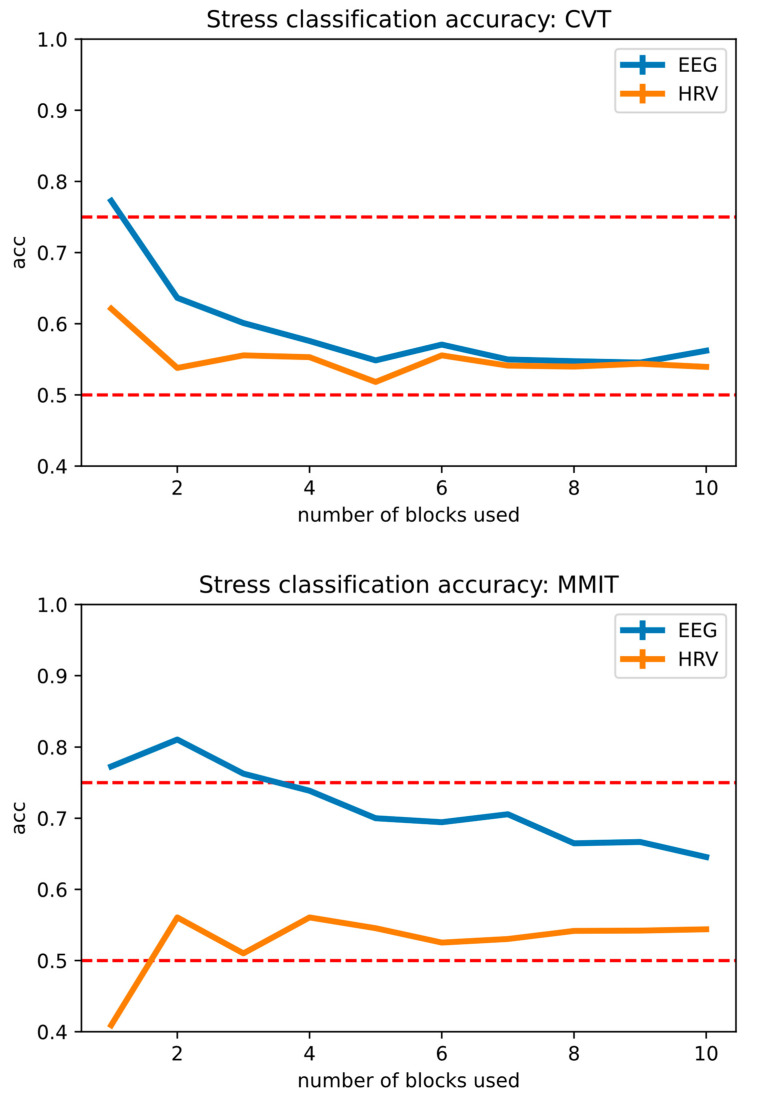
Stress detection accuracy for the CVT and MMIT, based on the model input and number of blocks used. Top: The accuracy of the stress-detection model built from CVT data. Bottom: The accuracy of the stress-detection model built from MMIT data. The x-axis represents the number of blocks from the start and end of the session used to label stress for training and testing, and the y-axis indicates classification accuracy. Reference accuracy levels of 50% (chance level) and 75% (minimum level for a useful brain-machine interface) are shown with red dotted lines.

Figure 8 below illustrates the performance of our stress detection model across varying numbers of task blocks, using the CVT and the MMIT, and either EEG or HRV as the input to the model.

As the HRV features used for classification are not standardized across studies [56], we used time-domain HRV features (SDNN, RMSSD, pNN50 and mean heart rate) that have been established for use in ultra-short-term measurements (<5 min) [56], as each of our blocks are approximately 1 min long.

For MMIT, the best classification accuracy was achieved at 2 blocks, at 81.0% for EEG features and 56.0% for HRV features, respectively. For CVT, the best classification accuracy was achieved at 1 block, at 77.2% for EEG features and 62.1% for HRV features, respectively. Adding a larger number of task blocks to the models decreased accuracy, suggesting that these blocks were contaminated with non-stress-related noise and should not be added to the model.

In both cases, our machine learning models trained from EEG features obtained higher classification accuracies than machine learning models trained from HRV features. We also observed that stress states induced by the MMIT were more detectable than stress states induced by the CVT, which lines up with our DSSQ survey results (see Figure 5). The overall performance of each stress detection model is summarized in Table 3 below.

## 4. Discussion

We developed EEG-based BCI models for detecting stress states and evaluated them by recording EEG signals from 40 participants who performed two complicated cognitive tasks. Our models performed to a high degree of accuracy when decoding stress states from EEG recordings; however, they fared poorly when decoding stress states from HRV recordings.

### 4.1. Inducing Stress with Cognitive Tasks

The CVT and MMIT are both complex tasks that require prolonged cognitive vigilance, and this is known to be a stressor [45,46]. Why, then, did our CVT not evoke significant levels of subjective stress in our participants? One possibility is that the duration was too short. Our participants were all Singaporean working adults, and Singapore is known for frequent cases of overwork [69]. Compared to typical 9-6 working hours, the 40 min CVT test period might have appeared to be mild, or even a relaxing break from work.

In contrast, the MMIT evoked significantly raised levels of distress in participants. We theorize that this is because the MMIT has a greater task complexity, requiring participants to read a list of conditions and apply them to each trial, rather than just reading a series of digit pairs. Also, the adaptive difficulty levels ensured that participants were “kept on their toes” and were always experiencing a difficulty level where they could not consistently respond to trials correctly. For the CVT, which had a fixed difficulty, participants could learn to perform the task better as they performed it, leading to a decrease in perceived difficulty over time, and therefore lower stress levels.

### 4.2. Decoding Stress with EEG Data

Our EEG-based stress decoder demonstrated good performance with both the CVT and MMIT training sets, with the MMIT data yielding a decoder that outperformed the CVT data. This aligns with our earlier observations that the MMIT evoked significant levels of distress, while the CVT did not. Nonetheless, both the CVT and MMIT stress states were decodable at a level of accuracy higher that 70%, well above the minimum level of accuracy required to create a useful binary decoder [70]. The flexibility of selective task blocks allows us to determine a suitable number of task blocks to achieve an optimal decoder.

### 4.3. Decoding Stress with HRV Data

Using HRV to predict the “stressed” or “not stressed” labels, we achieved a lower level of accuracy. This indicates that there is some feature in HRV for predicting stress levels, even though the accuracy is lower. Nonetheless, more work on this is needed, as this level of accuracy is insufficient for useful binary decoding [70].

Why was this the case, despite HRV being a well-known biomarker for stress [12,13]? One possibility is that there is a variation in individual stress responses that confounds our ability to decode stress from HRV. HRV is affected by cortisol levels (which are in turn downstream from the hypothalamic–pituitary–adrenal (HPA) axis), and it has been reported that people can have different patterns of cortisol and HRV in response to stress [71]. The stress response of the HPA axis is also affected by genetic, epigenetic, early-life environmental, and current environmental factors, all of which vary between participants [72,73].

### 4.4. Limitations and Future Directions

While our results were interesting and our models sufficiently accurate to build a stress-detection system, there are still many limitations in this research, which expand many possibilities for improvement in future studies. Perhaps, as stated in Section 4.1, there may not be enough stress, or too few types of stress, induced by our experimental tasks. Multi-component stressors such as the Trier Social Stress Test (TSST), Socially Evaluated Cold-Pressor Test (SECPT) or the Mannheim Multicomponent Stress Test (MMST) may evoke neural signals that are more readily decoded [13,31,39,40].

Alternatively, creating stressful situations in a controlled laboratory environment through a computer task may be unrealistic and not entirely feasible, as there are nearly no real-world consequences to failure for our cognitive tasks. Instead, stress is more likely to arise from threats perceived to be real, such as a risk of bodily harm, or the social threat from failing or being perceived to fail [43]. While it is unethical to expose experimental participants to real physical dangers, we can observe participants facing real-life social threats as they perform work at their regular workplaces. In a future study, we could recruit participants who work in high-pressure work environments and record EEG and HRV signals from them in situ. This may allow us to observe higher stress levels, and hence obtain better classification accuracies on stress.

Meanwhile, the end-goal of producing a real-time stress-detection system for people at work necessitated the use of dry EEG electrodes, yet our dry electrode system (Neeuro Senzeband 2) only has four data channels, and for several participants we recorded high levels of noise. To improve the data quality, we could focus on the hardware by purchasing a newer system, potentially with more electrodes, and/or focus on the data stream by implementing real-time signal quality monitoring and adaptive noise reduction techniques. Another possibility is to record more types of physiological signals, such as galvanic skin response, facial electromyography, respiration rate, and salivary cortisol levels, to produce more holistic and accurate models.

We recruited participants from a diverse range of ages (21–65), ethnicities and cultural backgrounds (including Chinese, Malay, Indian, Japanese, and Eurasian participants), and professions (including scientists, engineers, students, sales workers, and office workers).

Nonetheless, another potential limitation is that our 40 participants were drawn from a relatively small section of humanity, and therefore may not be generalizable to humanity as a whole. While Singapore is not Western, the other WEIRD factors (Western, Educated, Industrialized, Rich, and Democratic) described by Henrich et al. still apply to our participants in this study [74]. Future studies on stress detection may recruit a larger cohort of participants, and include participants drawn from different ages, cultural backgrounds, and professional fields. We could also potentially collaborate with researchers based in other countries to obtain a larger and more diverse sample of participants. These steps may help us to obtain a more comprehensive understanding of stress across different demographics.

Also, our results in Figure 5 suggest that the CVT was not stressful enough to cause significant increases in self-reported stress. This matches the results in Table 3, where our EEG model was not able to decode stressed blocks in the CVT as effectively as when the same model was applied to the MMIT. If we use the CVT to induce stress in future experiments, we will increase its stress level. Ways to do so include decreasing the duration of each trial, increasing the overall duration of the session, increasing the number of digit pairs presented during each trial, and/or implementing a dynamic difficulty setting like we did with the MMIT. For example, a participant who is performing well might be required to search for critical signals in a larger number of digit pairs displayed during each trial, or have their trial duration shortened.

After optimizing the number of blocks used for training our models, we found that using only 2 or 4 blocks from each participant produced the best classification accuracies. While this method minimizes the non-stress-related signals in the dataset, we recognize that this also removes most of the recordings from the models, potentially resulting in overfitting. Future studies perhaps could avoid this problem by frequently asking participants to self-report their stress levels via a Likert scale every few blocks of trials, allowing experimenters to obtain stress level labels from the middle of the recordings as well. Then, the entire recording for each participant can be fed into the stress classification model, as it would be accompanied by frequently updated stress labels.

HRV is known to be an established method for detecting stress in healthy adults [75], and has been used in conjunction with EEG to detect stress [27,76]. Nonetheless, our HRV-based stress classification model did not perform as well as our EEG-based model. One potential reason is that stress levels in participants may have been confounded by other factors such as fatigue, or a change in contact impedance due to perspiration. While our ECG sensor did not have the ability to monitor contact impedance, future studies may integrate improved sensors that do, and therefore reduce the possibility of such factors impacting the decoding of stress states. Another possibility is using both EEG and HRV features as inputs to stress detection models.

Additionally, to verify the reliability of the self-reported stress results, we can implement other forms of stress questionnaires, such as the Task Load Index (NASA-TLX) or a modified Perceived Stress Scale [77,78]. Longitudinal studies could also be conducted to monitor how the participants’ stress responses changed over time, e.g., if they were asked to perform the same task over multiple sessions on different days, to gain valuable insights into stress dynamics and resilience to stress.

Another approach we can take is to investigate the neurophysiological basis of stress: to study what is going on in the brain as stress levels increase. After all, EEG measures the summed activity of neurons in the brain, and can be used to make inferences on stress-related brain activity [13,79]. While microelectrode recordings are invasive and impractical to perform on human volunteers, there are other options such as Near-Infrared Spectroscopy (NIRS), which can be used to directly observe stress-related hemodynamic changes in the cortex using sensors placed over the skin of the scalp [80]. Alternatively, computational modeling can be used to predict how neural activity at the cellular level creates EEG at the macro-scale level [81]. Either way, a better understanding of how stress affects neural activity in the brain could be used to design more rational methods to detect and treat stress.

We also are in the process of planning a follow-up study where participants performing their work duties are monitored by an EEG system for several hours. We will pair the EEG system with regular questionnaires so participants can self-report their stress levels. This way, we can determine if our stress monitoring system is able to detect the correlates of real-life work stress in situ, not just stress artificially induced by an experimental task.

In addition, to mitigate the issue of block-to-block variability, we will see if predicted stress levels smoothed over several blocks (2 or more) empirically fit self-reported stress levels better. This way, we can produce a system that matches reported stress levels better.

Our follow-up study can also include more sophisticated forms of feature extraction such as wavelet transforms, Common Spatial Pattern (CSP)-based techniques, or artificial neural network (ANN)-based feature extraction [82,83,84]. We could also focus on non-linear feature extraction techniques for electrophysiological signals, such as improved symbolic aggregate approximation (isaxEDA), complexity analysis (comEDA), topological analysis (topEDA), and network theory-based analysis (netEDA) [61]. These may yield more accurate models, and produce more insights into the neurophysiology of cognitive stress, albeit at the cost of processing power.

## 5. Conclusions

As people perform mental tasks, stress levels can increase, reducing task performance. This can have adverse effects in workplaces which require staff to continuously shoulder heavy cognitive burdens. To monitor stress, we have created an EEG-based BCI system that can detect subjective mental stress in the context of two complicated cognitive tasks. Our system performs at a high level of accuracy, with an accuracy of 81.0% for MMIT, the more stressful task. This model can potentially be used to monitor stress levels in real-time at high-pressure workplaces. However, if stress is instead decoded from HRV recordings, our model fares far more poorly, with accuracy levels at 62.1% or lower. We propose that our EEG-based BCI system can effectively classify stress states in the context of people performing stressful cognitive tasks.

## Figures and Tables

**Table 1:** BCI experiment workflow. CVT—Cognitive Vigilance Task; MMIT—Multi-Modal Integration Task. The experiment consisted of 8 steps, indicated as S1–S8. EEG and ECG data were recorded in steps S3, S4, and S7 (blue background).

	S1	S2	S3	S4	S5	S6	S7	S8
50%	Preparation	Questionnaire Block 1	Baseline (calm video)	Task 1—CVT	Questionnaire Block 2	Rest	Task 2—MMIT	Questionnaire Block 4
50%	Preparation	Questionnaire Block 1	Baseline (calm video)	Task 1—MMIT	Questionnaire Block 2	Rest	Task 2—CVT	Questionnaire Block 4
	5 min	10 min	10 min	40 min	10 min	15 min	40 min	10 min

**Table 2:** List of HRV features that were extracted from the ECG data.

Feature	Description
mean_nni	Mean NN interval
Sdnn	Standard deviation of NN interval
Sdsd	Standard deviation of successive RR interval differences
Nni_50	Number of NN intervals that differ by more than 50 ms
Pnni_50	Percentage of successive RR intervals that differ by more than 50 ms
Nni_20	Number of NN intervals that differ by more than 20 ms
Pnni_20	Percentage of successive RR intervals that differ by more than 20 ms
rmssd	Root mean square of successive RR interval differences
median_nni	Median NN interval
range_nni	Range of NN intervals
cvsd	The root mean square of successive differences (RMSSD) divided by the mean of the RR intervals (MeanNN)
cvnni	The standard deviation of the RR intervals (SDNN) divided by the mean of the RR intervals (MeanNN).
mean_hr	Mean heart rate
max_hr	Max heart rate

**Table 3:** Mental stress model performance from the two tasks using the optimal number of blocks, mean across all participants. Values are expressed as mean ± SEM.

Task	EEG Decoding Accuracy	HRV Decoding Accuracy
MMIT	81.0 ± 3.4%	56.0 ± 4.3%
CVT	77.2 ± 5.2%	62.1 ± 6.0%

## Data Availability

The original data presented in the study are openly available in the Digital Repository of NTU (DR-NTU) at https://doi.org/10.21979/N9/ZJM6WF.

## References

[1] Borghini G., Bandini A., Orlandi S., Di Flumeri G., Arico P., Sciaraffa N., Ronca V., Bonelli S., Ragosta M., Tomasello P. Stress Assessment by Combining Neurophysiological Signals and Radio Communications of Air Traffic Controllers. Proceedings of the 2020 42nd Annual International Conference of the IEEE Engineering in Medicine & Biology Society (EMBC).

[2] Joseph M., Ray J.M., Chang J., Cramer L.D., Bonz J.W., Yang T.J., Wong A.H., Auerbach M.A., Evans L.V. (2022). All Clinical Stressors Are Not Created Equal: Differential Task Stress in a Simulated Clinical Environment. AEM Educ. Train..

[3] Kjellberg A., Toomingas A., Norman K., Hagman M., Herlin R.-M., Tornqvist E.W. (2010). Stress, Energy and Psychosocial Conditions in Different Types of Call Centres. Work.

[4] Arora S., Sevdalis N., Nestel D., Woloshynowych M., Darzi A., Kneebone R. (2010). The Impact of Stress on Surgical Performance: A Systematic Review of the Literature. Surgery.

[5] Luers P., Schloeffel M., Prüssner J.C. (2020). Working Memory Performance Under Stress. Exp. Psychol..

[6] Riddell C., Yonelinas A.P., Shields G.S. (2023). When Stress Enhances Memory Encoding: The Beneficial Effects of Changing Context. Neurobiol. Learn. Mem..

[7] Juster R.-P., McEwen B.S., Lupien S.J. (2010). Allostatic Load Biomarkers of Chronic Stress and Impact on Health and Cognition. Neurosci. Biobehav. Rev..

[8] Yaribeygi H., Panahi Y., Sahraei H., Johnston T.P., Sahebkar A. (2017). The Impact of Stress on Body Function: A Review. EXCLI J.

[9] Tomei G., Cinti M.E., Cerratti D., Fioravanti M. (2006). Attention, repetitive works, fatigue and stress. Ann. Ig..

[10] Roos L.E., Giuliano R.J., Beauchamp K.G., Berkman E.T., Knight E.L., Fisher P.A. (2020). Acute Stress Impairs Children’s Sustained Attention with Increased Vulnerability for Children of Mothers Reporting Higher Parenting Stress. Dev. Psychobiol..

[11] Ochiai Y., Takahashi M., Matsuo T., Sasaki T., Sato Y., Fukasawa K., Araki T., Otsuka Y. (2023). Characteristics of Long Working Hours and Subsequent Psychological and Physical Responses: JNIOSH Cohort Study. Occup. Environ. Med..

[12] Katmah R., Al-Shargie F., Tariq U., Babiloni F., Al-Mughairbi F., Al-Nashash H. (2021). A Review on Mental Stress Assessment Methods Using EEG Signals. Sensors.

[13] Vanhollebeke G., De Smet S., De Raedt R., Baeken C., van Mierlo P., Vanderhasselt M.-A. (2022). The Neural Correlates of Psychosocial Stress: A Systematic Review and Meta-Analysis of Spectral Analysis EEG Studies. Neurobiol. Stress.

[14] Föhr T., Tolvanen A., Myllymäki T., Järvelä-Reijonen E., Rantala S., Korpela R., Peuhkuri K., Kolehmainen M., Puttonen S., Lappalainen R. (2015). Subjective Stress, Objective Heart Rate Variability-Based Stress, and Recovery on Workdays among Overweight and Psychologically Distressed Individuals: A Cross-Sectional Study. J. Occup. Med. Toxicol..

[15] Christensen D.S., Dich N., Flensborg-Madsen T., Garde E., Hansen Å.M., Mortensen E.L. (2019). Objective and Subjective Stress, Personality, and Allostatic Load. Brain Behav..

[16] Shields G.S., Fassett-Carman A., Gray Z.J., Gonzales J.E., Snyder H.R., Slavich G.M. (2023). Why Is Subjective Stress Severity a Stronger Predictor of Health Than Stressor Exposure? A Preregistered Two-Study Test of Two Hypotheses. Stress Health.

[17] Rashid M., Sulaiman N., Abdul Majeed A.P.P., Musa R.M., Ab. Nasir A.F., Bari B.S., Khatun S. (2020). Current Status, Challenges, and Possible Solutions of EEG-Based Brain-Computer Interface: A Comprehensive Review. Front. Neurorobot..

[18] Yadav H., Maini S. (2023). Electroencephalogram Based Brain-Computer Interface: Applications, Challenges, and Opportunities. Multimed. Tools Appl..

[19] Lim R.Y., Lew W.-C.L., Ang K.K. (2024). Review of EEG Affective Recognition with a Neuroscience Perspective. Brain Sci..

[20] Lopez-Gordo M.A., Sanchez-Morillo D., Valle F.P. (2014). Dry EEG Electrodes. Sensors.

[21] Pope L. (2013). Papers, Please. https://papersplea.se/.

[22] McKernan B. (2021). Digital Texts and Moral Questions About Immigration: Papers, Please and the Capacity for a Video Game to Stimulate Sociopolitical Discussion. Games Cult..

[23] Sievers J.M. (2020). Papers, Please as Philosophy. The Palgrave Handbook of Popular Culture as Philosophy.

[24] Sussman R.F., Sekuler R. (2022). Feeling Rushed? Perceived Time Pressure Impacts Executive Function and Stress. Acta Psychol..

[25] Szalma J.L., Teo G.W.L. (2012). Spatial and Temporal Task Characteristics as Stress: A Test of the Dynamic Adaptability Theory of Stress, Workload, and Performance. Acta Psychol..

[26] Claypoole V.L., Dever D.A., Denues K.L., Szalma J.L. (2019). The Effects of Event Rate on a Cognitive Vigilance Task. Hum. Factors.

[27] Attar E.T., Balasubramanian V., Subasi E., Kaya M. (2021). Stress Analysis Based on Simultaneous Heart Rate Variability and EEG Monitoring. IEEE J. Transl. Eng. Health Med..

[28] Perez-Valero E., Vaquero-Blasco M.A., Lopez-Gordo M.A., Morillas C. (2021). Quantitative Assessment of Stress Through EEG During a Virtual Reality Stress-Relax Session. Front. Comput. Neurosci..

[29] Badr Y., Al-Shargie F., Tariq U., Babiloni F., Al Mughairbi F., Al-Nashash H. Classification of Mental Stress Using Dry EEG Electrodes and Machine Learning. Proceedings of the 2023 Advances in Science and Engineering Technology International Conferences (ASET).

[30] Bali A., Jaggi A.S. (2015). Clinical Experimental Stress Studies: Methods and Assessment. Rev. Neurosci..

[31] Kirschbaum C., Pirke K.-M., Hellhammer D.H. (1993). The “Trier Social Stress Test”: A Tool for Investigating Psychobiological Stress Responses in a Laboratory Setting. Neuropsychobiology.

[32] Allen A.P., Kennedy P.J., Dockray S., Cryan J.F., Dinan T.G., Clarke G. (2016). The Trier Social Stress Test: Principles and Practice. Neurobiol. Stress.

[33] Brouwer A.-M., Hogervorst M.A. (2014). A New Paradigm to Induce Mental Stress: The Sing-a-Song Stress Test (SSST). Front. Neurosci..

[34] van der Mee D.J., Duivestein Q., Gevonden M.J., Westerink J.H.D.M., de Geus E.J.C. (2020). The Short Sing-a-Song Stress Test: A Practical and Valid Test of Autonomic Responses Induced by Social-Evaluative Stress. Auton. Neurosci..

[35] Hines E.A., Brown G.E. (1936). The Cold Pressor Test for Measuring the Reactibility of the Blood Pressure: Data Concerning 571 Normal and Hypertensive Subjects. Am. Heart J..

[36] Lamotte G., Boes C.J., Low P.A., Coon E.A. (2021). The Expanding Role of the Cold Pressor Test: A Brief History. Clin. Auton. Res..

[37] Fyer M.R., Uy J., Martinez J., Goetz R., Klein D.F., Fyer A., Liebowitz M.R., Gorman J. (1987). CO2 Challenge of Patients with Panic Disorder. Am. J. Psychiatry.

[38] Vickers K., Jafarpour S., Mofidi A., Rafat B., Woznica A. (2012). The 35% Carbon Dioxide Test in Stress and Panic Research: Overview of Effects and Integration of Findings. Clin. Psychol. Rev..

[39] Minkley N., Schröder T.P., Wolf O.T., Kirchner W.H. (2014). The Socially Evaluated Cold-Pressor Test (SECPT) for Groups: Effects of Repeated Administration of a Combined Physiological and Psychological Stressor. Psychoneuroendocrinology.

[40] Kolotylova T., Koschke M., Bär K.-J., Ebner-Priemer U., Kleindienst N., Bohus M., Schmahl C. (2010). [Development of the “Mannheim Multicomponent Stress Test” (MMST)]. Psychother. Psychosom. Med. Psychol..

[41] Scarpina F., Tagini S. (2017). The Stroop Color and Word Test. Front. Psychol..

[42] Warm J. (1984). Sustained Attention in Human Performance.

[43] Craw O.A., Smith M.A., Wetherell M.A. (2021). Manipulating Levels of Socially Evaluative Threat and the Impact on Anticipatory Stress Reactivity. Front. Psychol..

[44] Von Baeyer C.L., Piira T., Chambers C.T., Trapanotto M., Zeltzer L.K. (2005). Guidelines for the Cold Pressor Task as an Experimental Pain Stimulus for Use with Children. J. Pain.

[45] Warm J.S., Parasuraman R., Matthews G. (2008). Vigilance Requires Hard Mental Work and Is Stressful. Hum. Factors.

[46] Dillard M.B., Warm J.S., Funke G.J., Nelson W.T., Finomore V.S., McClernon C.K., Eggemeier F.T., Tripp L.D., Funke M.E. (2019). Vigilance Tasks: Unpleasant, Mentally Demanding, and Stressful Even When Time Flies. Hum. Factors.

[47] Meule A. (2017). Reporting and Interpreting Task Performance in Go/No-Go Affective Shifting Tasks. Front. Psychol..

[48] Matthews G., Campbell S.E., Falconer S., Joyner L.A., Huggins J., Gilliland K., Grier R., Warm J.S. (2002). Fundamental Dimensions of Subjective State in Performance Settings: Task Engagement, Distress, and Worry. Emotion.

[49] Matthews G., Szalma J., Panganiban A.R., Neubauer C., Warm J.S. (2013). Profiling Task Stress With The Dundee State Questionnaire. Psychology of Stress: New Research.

[50] Vaessen T., Rintala A., Otsabryk N., Viechtbauer W., Wampers M., Claes S., Myin-Germeys I. (2021). The Association between Self-Reported Stress and Cardiovascular Measures in Daily Life: A Systematic Review. PLoS ONE.

[51] Jhangiani R.S., Cuttler C., Leighton D.C. (2019). Research Methods in Psychology.

[52] Dalmeida K.M., Masala G.L. (2021). HRV Features as Viable Physiological Markers for Stress Detection Using Wearable Devices. Sensors.

[53] Bagliani G., Della Rocca D.G., De Ponti R., Capucci A., Padeletti M., Natale A. (2018). Ectopic Beats: Insights from Timing and Morphology. Card. Electrophysiol. Clin..

[54] Acar B., Savelieva I., Hemingway H., Malik M. (2000). Automatic Ectopic Beat Elimination in Short-Term Heart Rate Variability Measurement. Comput. Methods Programs Biomed..

[55] Aura-Healthcare/Hrv-Analysis. Package for Heart Rate Variability Analysis in Python. https://github.com/Aura-healthcare/hrv-analysis.

[56] Shaffer F., Ginsberg J.P. (2017). An Overview of Heart Rate Variability Metrics and Norms. Front. Public Health.

[57] Richman J.S., Moorman J.R. (2000). Physiological Time-Series Analysis Using Approximate Entropy and Sample Entropy. Am. J. Physiol. Heart Circ. Physiol..

[58] Higuchi T. (1988). Approach to an Irregular Time Series on the Basis of the Fractal Theory. Phys. D Nonlinear Phenom..

[59] Hjorth B. (1970). EEG Analysis Based on Time Domain Properties. Electroencephalogr. Clin. Neurophysiol..

[60] Stam C.J. (2005). Nonlinear Dynamical Analysis of EEG and MEG: Review of an Emerging Field. Clin. Neurophysiol..

[61] Veeranki Y.R., Diaz L.R.M., Swaminathan R., Posada-Quintero H.F. (2024). Nonlinear Signal Processing Methods for Automatic Emotion Recognition Using Electrodermal Activity. IEEE Sens. J..

[62] Liang Z., Wang Y., Sun X., Li D., Voss L.J., Sleigh J.W., Hagihira S., Li X. (2015). EEG Entropy Measures in Anesthesia. Front. Comput. Neurosci..

[63] Akar S.A., Kara S., Agambayev S., Bilgic V. Nonlinear Analysis of EEG in Major Depression with Fractal Dimensions. Proceedings of the 2015 37th annual international conference of the IEEE Engineering in Medicine and Biology Society (EMBC).

[64] Ruiz-Padial E., Ibáñez-Molina A.J. (2018). Fractal Dimension of EEG Signals and Heart Dynamics in Discrete Emotional States. Biol. Psychol..

[65] Vicchietti M.L., Ramos F.M., Betting L.E., Campanharo A.S.L.O. (2023). Computational Methods of EEG Signals Analysis for Alzheimer’s Disease Classification. Sci. Rep..

[66] Veeranki Y.R., Ganapathy N., Swaminathan R. (2021). Non-Parametric Classifiers Based Emotion Classification Using Electrodermal Activity and Modified Hjorth Features. Stud. Health Technol. Inf..

[67] Hag A., Al-Shargie F., Handayani D., Asadi H. (2023). Mental Stress Classification Based on Selected Electroencephalography Channels Using Correlation Coefficient of Hjorth Parameters. Brain Sci..

[68] Foong R., Ang K.K., Zhang Z., Quek C. (2019). An Iterative Cross-Subject Negative-Unlabeled Learning Algorithm for Quantifying Passive Fatigue. J. Neural Eng..

[69] Wong K., Chan A.H.S., Ngan S.C. (2019). The Effect of Long Working Hours and Overtime on Occupational Health: A Meta-Analysis of Evidence from 1998 to 2018. Int J Env. Res Public Health.

[70] Vidaurre C., Blankertz B. (2010). Towards a Cure for BCI Illiteracy. Brain Topogr..

[71] Bennett M.M., Tomas C.W., Fitzgerald J.M. (2024). Relationship between Heart Rate Variability and Differential Patterns of Cortisol Response to Acute Stressors in Mid-life Adults: A Data-driven Investigation. Stress Health.

[72] Stephens M.A.C., Wand G. (2012). Stress and the HPA Axis. Alcohol. Res..

[73] Meaney M.J., Szyf M. (2005). Environmental Programming of Stress Responses through DNA Methylation: Life at the Interface between a Dynamic Environment and a Fixed Genome. Dialogues Clin. Neurosci..

[74] Henrich J., Heine S.J., Norenzayan A. (2010). The Weirdest People in the World?. Behav. Brain Sci..

[75] Immanuel S., Teferra M.N., Baumert M., Bidargaddi N. (2023). Heart Rate Variability for Evaluating Psychological Stress Changes in Healthy Adults: A Scoping Review. Neuropsychobiology.

[76] Hemakom A., Atiwiwat D., Israsena P. (2023). ECG and EEG Based Detection and Multilevel Classification of Stress Using Machine Learning for Specified Genders: A Preliminary Study. PLoS ONE.

[77] Hart S.G., Staveland L.E. (1988). Development of NASA-TLX (Task Load Index): Results of Empirical and Theoretical Research. Human Mental Workload.

[78] Chan S.F., La Greca A.M., Gellman M.D., Turner J.R. (2013). Perceived Stress Scale (PSS). Encyclopedia of Behavioral Medicine.

[79] Rho G., Callara A.L., Bernardi G., Scilingo E.P., Greco A. (2023). EEG Cortical Activity and Connectivity Correlates of Early Sympathetic Response during Cold Pressor Test. Sci. Rep..

[80] Chou P.-H., Lin W.-H., Hung C.-A., Chang C.-C., Li W.-R., Lan T.-H., Huang M.-W. (2016). Perceived Occupational Stress Is Associated with Decreased Cortical Activity of the Prefrontal Cortex: A Multichannel Near-Infrared Spectroscopy Study. Sci. Rep..

[81] Martínez-Cañada P., Ness T.V., Einevoll G.T., Fellin T., Panzeri S. (2021). Computation of the Electroencephalogram (EEG) from Network Models of Point Neurons. PLoS Comput. Biol..

[82] Daubechies I. (1992). Ten Lectures on Wavelets: 61.

[83] Ang K.K., Chin Z.Y., Zhang H., Guan C. Filter Bank Common Spatial Pattern (FBCSP) in Brain-Computer Interface. Proceedings of the 2008 IEEE International Joint Conference on Neural Networks (IEEE World Congress on Computational Intelligence).

[84] Singh A.K., Krishnan S. (2023). Trends in EEG Signal Feature Extraction Applications. Front. Artif. Intell..

